# Simple use of Y-connector for proper positioning and contrast aspiration in biliary metal stent placement

**DOI:** 10.1055/a-2225-5570

**Published:** 2024-01-09

**Authors:** Kiyoyuki Kobayashi, Hideki Kobara, Takako Nomura, Tomohiro Ogi, Hideki Kamada, Masafumi Ono, Tsutomu Masaki

**Affiliations:** 1 Division of Innovative Medicine for Hepatobiliary and Pancreatology, Kagawa University, Faculty of Medicine, Kagawa, Japan; 2 Department of Gastroenterology and Hepatology, HITO Medical Center, Ehime, Japan; 3 Department of Gastroenterology and Neurology, Kagawa University, Faculty of Medicine, Kagawa, Japan

Endoscopic biliary drainage for unresectable malignant hilar biliary obstruction often requires multiple metal stents, and stent-in-stent placement is an effective drainage method 1 2 . Although multiple stent placement requires more cholangiograms for proper positioning, post-endoscopic retrograde cholangiopancreatography (ERCP) cholangitis remains a concern. Moreover, the over-injection of contrast medium and residual contrast medium increase post-ERCP cholangitis 3 4 5 . To overcome these issues, we introduced a simple method using a Y-connector attached to an existing metal stent ( Fig. 1 ).

**Fig. 1:**
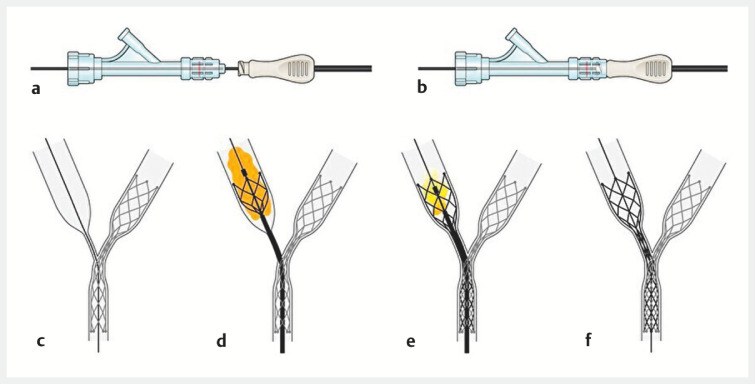
Schema showing attachment of Y-connector and stent device, contrast medium injection/aspiration. **a,b** The Y-connector is attached by rotating the distal end of the stent device and the Y-connector. The stent can also be attached with the guidewire threaded through the stent device. **c** For stent-in-stent placement, a guidewire and a second stent device with Y-connector are inserted into the target bile duct after the first stenting. **d** Because contrast using the Y-connector enabled visualization of the stenosis, it is easy to determine the position of the stent. **e** After stent placement, the stent device permits contrast medium aspiration with the guidewire in place to prevent cholangitis caused by the over-injection of contrast medium. **f** This method allows stenting while confirming proper stent placement and prevents post-endoscopic retrograde cholangiopancreatography (ERCP) cholangitis by aspirating as much contrast as possible. Source: Davinch Medical Illustration Office.

A 70-year-old woman undergoing chemotherapy for intrahepatic cholangiocarcinoma after a previous cholecystitis-associated endoscopic ultrasound-guided gallbladder drainage presented with obstructive jaundice for hilar biliary obstruction. Enhanced computed tomography revealed bilateral intra-bile duct dilation ( Fig. 2 ). Stent-in-stent placement was performed. The first metal stent was placed, and a guidewire was inserted through the mesh gap into the bile duct, where the second stent placement was intended. By this point, most of the contrast medium had leaked out, making the length of the stenosis and the target placement position difficult to determine ( Fig. 3 **a,b** ). A Y-connector (Access-9TM, Hemostasis Valve; SHEEN MAN Co., Ltd., Osaka, Japan) was attached to the proximal end of the guidewire lumen of the second stent device. The stent was inserted into the bile duct through a guidewire using a Y-connector. Because contrast using the Y-connector enabled visualization of the stenosis, it was easy to determine the position of the stent ( Fig. 3 **c** ). Furthermore, to prevent postoperative cholangitis after deployment, the remaining contrast medium could be aspirated easily without switching to a catheter ( Fig. 3 **d,e** ) ( Video 1 ). No post-ERCP cholangitis or elevated hepatobiliary enzyme levels were observed in this patient.

The combination of the stent device and Y-connector is a convenient and an efficacious method for confirming the stent position and preventing post-ERCP cholangitis.

**Fig. 2:**
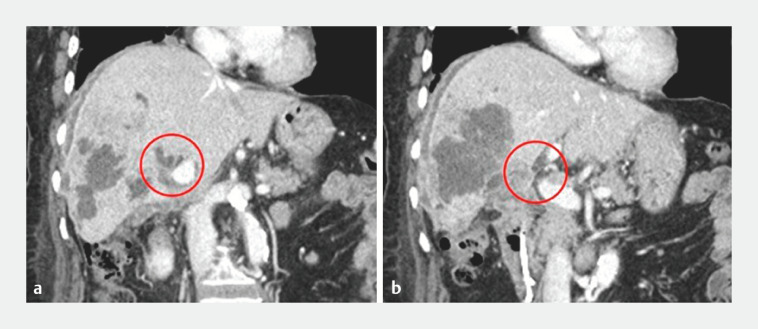
Enhanced computed tomography reveals bilateral intra-bile duct dilation.

**Fig. 3:**
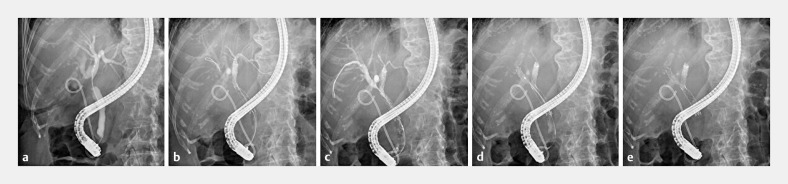
Endoscopic retrograde cholangiography images. **a** Endoscopic retrograde cholangiography reveals bilateral intra-bile duct dilation. **b** Although the first stent was placed in the left intrahepatic bile duct, most of the contrast medium had leaked out, making the length of the stenosis and the target placement position difficult to determine. **c** Since contrast using the Y-connector enabled the visualization of the stenosis, the position of the stent was easy to determine. **d** The remaining contrast agent could be aspirated easily without switching to a catheter. **e** No post-ERCP cholangitis and elevated hepatobiliary enzymes occurred.

Video showing the simple use of the Y-connector for proper positioning and the prevention of post-endoscopic retrograde cholangiopancreatography cholangitis during biliary metal stent placement.Video 1

Endoscopy_UCTN_Code_TTT_1AR_2AZ

## Notes

Endoscopy E-Videos https://eref.thieme.de/e-videos
*E-Videos* is an open access online section of the journal *Endoscopy* , reporting on interesting cases and new techniques in gastroenterological endoscopy. All papers include a high-quality video and are published with a Creative Commons CC-BY license. Endoscopy E-Videos qualify for HINARI discounts and waivers and eligibility is automatically checked during the submission process. We grant 100% waivers to articles whose corresponding authors are based in Group A countries and 50% waivers to those who are based in Group B countries as classified by Research4Life (see: https://www.research4life.org/access/eligibility/ ). This section has its own submission website at https://mc.manuscriptcentral.com/e-videos .
